# Development of a new broad-spectrum microencapsulation-based spray drying formulation of *Bacillus thuringiensis* subsp. *kurstaki* IMBL-B9 for the control of moths

**DOI:** 10.3389/fmicb.2023.1273725

**Published:** 2023-11-22

**Authors:** Kalaiselvi Duraisamy, Nan Hee Yu, Seon Hwa Kim, Jong Hwi Baek, Ji Yeon Son, Euna Choi, Min Gu Park, Jiwon Kim, Jae Young Choi, Mee Kyung Sang, Yeon Ho Je, Jin-Cheol Kim

**Affiliations:** ^1^Plant Healthcare Research Institute, JAN153 Biotech Incorporated, Gwangju, Republic of Korea; ^2^Research Institute for Agriculture and Life Sciences, Seoul National University, Seoul, Republic of Korea; ^3^Division of Agricultural Microbiology, National Institute of Agricultural Sciences, Rural Development Administration, Wanju, Republic of Korea; ^4^Department of Agricultural Biotechnology, College of Agriculture and Life Sciences, Seoul National University, Seoul, Republic of Korea; ^5^Department of Agricultural Chemistry and Institute of Environmentally Friendly Agriculture, College of Agriculture and Life Sciences, Chonnam National University, Gwangju, Republic of Korea

**Keywords:** biological control, *Bacillus thuringiensis*, Btk IMBL-B9, microencapsulation, spray drying, insecticidal activity

## Abstract

Certain *Bacillus thuringiensis* (Bt) strains such as Bt subsp. *kurstaki* and Bt subsp. *aizawai* have been widely used for pest management in agricultural practices. However, each strain only shows high specificity for pest control against a narrow range of lepidopteran species, and numerous lepidopteran pests have developed resistance to commercialized Bt strains. Therefore, there is a need for the development of novel Bt bioinsecticides which allow for potent and broad-spectrum insecticidal activity against lepidopteran species, including *Spodoptera* spp. (Noctuidae) and *Plutella xylostealla* (Plutellidae). In order to develop a novel bioinsecticide using Bt subsp. *kurstaki* IMBL-B9 (Btk IMBL-B9) that exhibits excellent insecticidal activity against three different lepidopteran species, we have developed a viable microencapsulation-based spray drying Btk IMBL-B9 formulation. The spore-crystal complex of Btk IMBL-B9 was microencapsulated using coating materials such as gum arabic, maltodextrin, and corn starch via spray drying. The encapsulated formulation of Btk IMBL-B9 presented an increased survival rate and storage stability at 54 ± 2°C for up to 6 weeks. The formulation showed similar insecticidal activity as the commercial bioinsecticide XenTari^®^ against *P. xylostella*. Under controlled greenhouse conditions, the Btk IMBL-B9 formulation was more effective against Lepidoptera spp. *S. frugiperda* and *P. xylostella*, than XenTari^®^. These results suggest that the microencapsulation-based spray drying formulation of Btk IMBL-B9 can be used effectively for the control of a wide range of moths.

## Introduction

The significance of novel agricultural developments in pest control extends beyond just crop protection. Effective pest control strategies can lead to increased crop yields, reduced crop losses, and improved food quality, thereby increasing food production and contributing to food security. The use of environmentally friendly pest control strategies, particularly through biological agents, can significantly reduce the use of harmful chemicals and pesticides, subsequently minimizing environmental pollution, input costs, and adverse health effects (Rusch et al., 2010). In recent years, there has been a marked rise in the research and development of biological pest control agents, resulting in the emergence of numerous novel products in the market, including microorganism-based pesticides (Ayilara et al., 2023). Among these microbial biopesticides, *Bacillus thuringiensis* (Bt), a spore-forming, gram-positive entomopathogenic bacterium, has been predominantly used due to its high specificity and low impact on non-target organisms. This versatile bacterium produces several insecticidal crystal proteins including Cry, Cyt, and Vip, which are encoded by *cry*, *cyt*, and *vip* genes, respectively (Palma et al., 2014). Upon solubilization and proteolytic activation in the insect midgut, these proteins are converted into active toxins that disrupt midgut membrane integrity, ultimately causing insect mortality (Gill et al., 1992).

Bt-based bioinsecticides have been available commercially since the late 1920s, however, their use is limited due to their narrow host spectrum, a short shelf life, and the development of pest resistance (Chandrasena et al., 2018). To address these challenges, researchers have collected Bt samples from various locations globally to isolate novel strains with broad host spectra and high insecticidal activity (Nair et al., 2018; Cao et al., 2020; Khorramnejad et al., 2020; Yin et al., 2020). Despite these efforts, there remains a shortage of Bt strains with both high toxicity and an expended host range, thus highlighting the need for novel insecticidal proteins which address these shortcomings. In 2021, a novel Bt strain, Bt subsp. *kurstaki* IMBL-B9 (Btk IMBL-B9), was isolated and evaluated for its biocontrol potential against polyphagous insect pests: *Spodoptera frugiperda*, *Spodoptera exigua* (Noctuidae) and *Plutella xylostella* (Plutellidae) (Park et al., 2022). The Btk IMBL-B9 strain harbours a total of eight crystal protein genes, including *cry1Aa*, *cry1Ac*, *cry1Be*, *cry1Ha*, *cry2Aa*, *cry2Ab*, *cry2Ah*, *cry1Ea*, and one vegetative insecticidal protein gene, *vip3Aa*. According to Park et al. (2022), this Bt strain exhibited excellent insecticidal activity against lepidopteran pests, with LC_50_ values comparable to those of the positive control strains Bt subsp. *aizawai* NT0423 (Bta NT0423) and Bt subsp. *kurstaki* HD-1 (Btk HD-1). In this study, the LC_50_ values (10^5^ CFU mL^−1^) of Btk IMBL-B9 against *S. frugiperda*, *S. exigua*, and *P. xylostella* were 4.9-fold, 5.6-fold and 34.7-fold lower than those of Bta NT0423, and 19.3-fold, 21.8-fold and 1.4-fold lower than those of the Btk HD-1 strain, respectively (Park et al., 2022). These observations suggest that the Btk IMBL-B9 strain has the potential to be used as a broad-spectrum microbial biopesticide for controlling lepidopteran pests.

Despite being considered the leading microbial bioinsecticide, the efficacy of Bt on foliar surfaces is affected by abiotic factors such as UV irradiation because the persistence of Bt crystal against insect pests are deactivated (Pozsgay et al., 1987; Sansinenea et al., 2015). Various Bt formulations have been developed to overcome these disadvantages and improve the shelf life, stability, and persistence of parasporal crystals in the environment. The development of optimum formulation of Bt is a crucial requirement for their effective commercial application. Microencapsulation-based spray drying formulation has garnered significant attention as a means of protecting Bt from environmental factors, as it allows for a controlled rate of release of insecticidal proteins (Bashir et al., 2016). Encapsulating agents or coating materials (natural or synthetic polymers) such as starch, maltodextrin, gum arabic, whey protein, or cellulose derivatives, form a protective shell around droplets containing the active ingredient, which prevents damage from heat stress during spray drying. Of these coating materials, maltodextrin, which is hydrolyzed from corn starch, is widely recognized as an excellent encapsulating agent due to their low viscosity and high water solubility (Xiao et al., 2022). Gum arabic is the most commonly used coating material in spray drying and is a natural composite of proteins and polysaccharides sourced from *Acacia* trees. It has a highly branched polysaccharide structure consisting of a galactose backbone with branches of rhamnose and arabinose, as well as a protein-arabinogalactan complex as a minor component (Dror et al., 2006).

In the present study, we have attempted to develop a microencapsulation-based spray drying formulation using Btk IMBL-B9, which allows for strong insecticidal activity against three different moth species, including *S. frugiperda*, *S. exigua*, and *P. xylostella.* Therefore, the objectives of this study are (1) to develop a microencapsulation-based spray drying formulation of Bkt IMBL-B9, using a mixture of gum arabic, corn starch and maltodextrin, (2) to measure the spore survival rate and storage stability of the formulation, (3) to investigate *in vitro* insecticidal activity, and (4) to evaluate the pest control efficacy of the formulation under greenhouse conditions.

## Materials and methods

### Fermentation of Btk IMBL-B9

A single colony of Btk IMBL*-*B9 was transferred into a 250-mL Erlenmeyer flask containing 50 mL of tryptic soy broth (TSB; Becton Dickinson and Company, ML, United States) medium which was then incubated at 30°C, at 220 rpm for 24 h to produce the seed culture. The 1% (v/v) seed culture was inoculated into a bioreactor (Pilot Scale Fermenter 50 L, Biocns Co., Ltd., Daejeon, Republic of Korea) containing 12 L of sterile medium B (15 g glucose, 10 g soybean meal, 2 g yeast extract, 2 g peptone, 0.3 g MgSO_4_ 7H_2_O, 0.02 g ZnSO_4_ 7H_2_O, 0.02 g FeSO_4_ 7H_2_O, 1 L distilled water) (Dulmage et al., 1970). Thereafter, fermentation was conducted using ‘batch mode’, along with the following conditions: temperature of 30°C, at 500 rpm, and an aeration rate of 1 vvm. Antifoam 204 (Sigma-Aldrich, Seoul, Korea) was used at a concentration of 0.7% as an antifoam agent. Fermentation was continued for 96 h to complete the sporulation and autolysis phase.

### Preparation of Btk IMBL-B9 spore-crystal complex

The spore-crystal complex from the fermentation broth was collected and concentrated 10-fold, using a Tubular Separator Centrifuge (A/T1250, Hanin Sci-Med Co., Ltd., Daejeon, Republic of Korea) at 14,000 rpm. Thereafter, a 15% coating materials mixture comprising gum Arabic, maltodextrin, and corn starch (Sigma-Aldrich, Seoul, Korea), was added to 1 L of spore-crystal complex (10^10^ colony forming unit (CFU) g^−1^) at a 1:1:1 ratio. The mixture was then continuously stirred using a laboratory stirrer (MS5050D; Misung S&I, Daejeon, Republic of Korea) at 300 rpm for 20 min.

### Microencapsulation-based spray drying formulation

Microencapsulation of the spore-crystal complex was performed using laboratory spray drying equipment (KLSD-1500, Koreamedi Co., Ltd., Daegu, Republic of Korea). The mixture of coating materials and spore-crystal complex suspension, as well as a spore-crystal complex without coating materials, were separately pumped into the drying chamber at a flow rate of 7 mL min^−1^. Following this, drying was performed at an inlet and outlet temperature of 140°C and 80–90°C, respectively. In the case of non-encapsulated Btk IMBL-B9 sample, no additive was incorporated. The encapsulated and non-encapsulated spray-dried formulations of Btk IMBL-B9 were collected from a cyclone and transferred to airtight containers, then stored for further analysis.

### Field emission scanning electron microscopy (FESEM)

The external appearance of the encapsulated and non-encapsulated spray drying Btk IMBL-B9 powder was evaluated using FESEM (FESEM HITACHI S-4800). The two samples were fixed in stubs using sticky carbon paper and coated with platinum.

### Spore survival rate after spray drying

The spore survival rate was determined as the fraction of viable spores after spray drying, over the viable spores before spray drying (Pungrasmi et al., 2019). The spore survival rate was expressed using the following equation.


$$\mathrm{Survival}\;\mathrm{rate}\;\left(\%\right)=\frac{N}{N_{0}}\times 100$$

Where *N* describes the logarithm of CFU g^−1^ after the spray drying process, and *N*_0_ describes the logarithm of CFU g^−1^ before the spray drying process.

### Storage stability

Two spray-dried samples were stored at 54 ± 2°C for 6 weeks. Evaluation of the spore viability was performed at 0, 2, 4, and 6 weeks after the spray drying process. One gram of spray-dried powder was dispersed in a 9 mL phosphate-buffered saline (PBS) solution and stirred for 3 min. Following this, these solutions were used to create serial dilutions, then plated on a ½ Nutrient agar (NA; Becton, Dickinson and Company) medium and incubated overnight. The spore viability was equated to the survival rate of spores after spray drying.

### SDS-PAGE analysis

In order to compare the crystal protein contents of the encapsulated formulation of Btk IMBL-B9 with those of the commercial bioinsecticide XenTari^®^ (Biological insecticide containing *Bt aizawai* ABTS-1857), 10 mg of powder was suspended in a 10 mL 1× PBS solution and stirred for 1 min. The samples were then mixed with Laemmli sample buffer and boiled for 5 min. The insecticidal crystal protein was then analysed on a 12%–15% SDS-PAGE gel (Laemmli, 1970).

### Insects

*Plutella xylostella* was obtained from the Environment-Friendly Agricultural Research Institute, Jeollanam-do Agricultural Research & Extension Services (Naju-si, Republic of Korea) and is adapted for breeding on rapeseed (*Brassica napus*). *S. frugiperda* was obtained from Jeon-Buk National University (Jeonju, Jeollabuk-do, Republic of Korea), and both are adapted to breeding on an artificial diet as previously reported by Park et al. (2022). All larvae used in this study were reared in a growth chamber at 25°C with 70% relative humidity and a 16 h light/8 h dark cycle.

### Indoor bioassay

The third-instar larvae of *P. xylostella* were subjected to the encapsulated and non-encapsulated spray drying formulations of Btk IMBL-B9 which stored at 54°C for 6 weeks, in order to determine insecticidal activity following a method set up by Park et al. (2022). XenTari^®^ stored at 54°C for 6 weeks was used as a positive control. Briefly, 10 third-instar larvae of *P. xylostella* were fed 5 × 5 cm Chinese cabbage leaves dipped in the two formulations of Btk IMBL-B9 and XenTari^®^ at 100,000- and 200,000-fold dilutions. The larval mortality was recorded at 48 h after treatment, and the treatments were independently replicated three times.

### Greenhouse study

A controlled greenhouse experiment was conducted using Chinese cabbage and cucumber plants from the Rural Development Administration in a completely randomized design with two replications, using three plants per replication. The six-leaf stage Chinese cabbage plants and four-leaf stage cucumber plants were treated with a 1,000-fold dilution of encapsulated formulation of Btk IMBL-B9 and XenTari^®^. Following this, 20 third-instar larvae of *P. xylostella* and *S. frugiperda* were introduced into in each Chinese cabbage plant pot, and 20 third-instar larvae of *S. frugiperda* were introduced into in each cucumber plant pot. After 3 days, the Chinese cabbage and cucumber plants were treated again with a 1,000-fold dilution of encapsulated formulation of Btk IMBL-B9 and XenTari^®^. The results were observed 7 days after the first spraying treatment.

### Statistical analysis

Statistical analysis was performed by one-way ANOVA using SPSS Statistics version 20 (SPSS Inc., Chicago, IL, United States). Multiple comparisons of mean values were calculated by *post-hoc* tests using Scheffé’s method. *p* values less than 0.05 were considered statistically significant.

## Results

### Formulation of encapsulated Btk IMBL-B9

**Figure 1:**
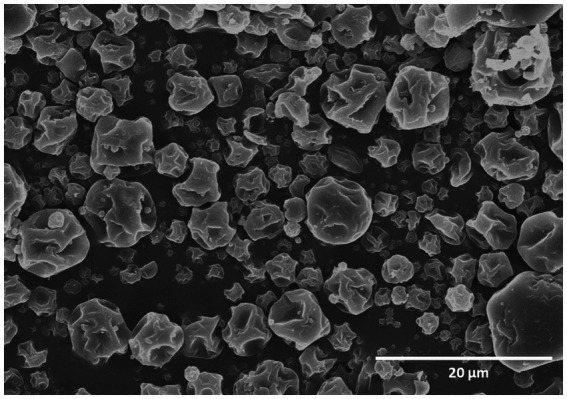
Field emission scanning electron microscope micrograph of the encapsulated Bt-IMBL-B9 particles.

In the current study, the development of a Btk IMBL-B9 formulation was performed using the spray drying method. In order to protect and/or increase the viability of Btk IMBL-B9 spores after spray drying, the spores were encapsulated using coating materials: gum arabic, maltodextrin and corn starch. The morphology of the encapsulated Btk IMBL-B9 powder was analysed by FESEM. As shown in Figure 1, the microencapsulation-based spray-dried particles were found to exhibit a partially collapsed small spherical shape, and varied in size ranging from 5 to 7 μm.

### Stability of encapsulated Btk IMBL-B9

**Figure 2:**
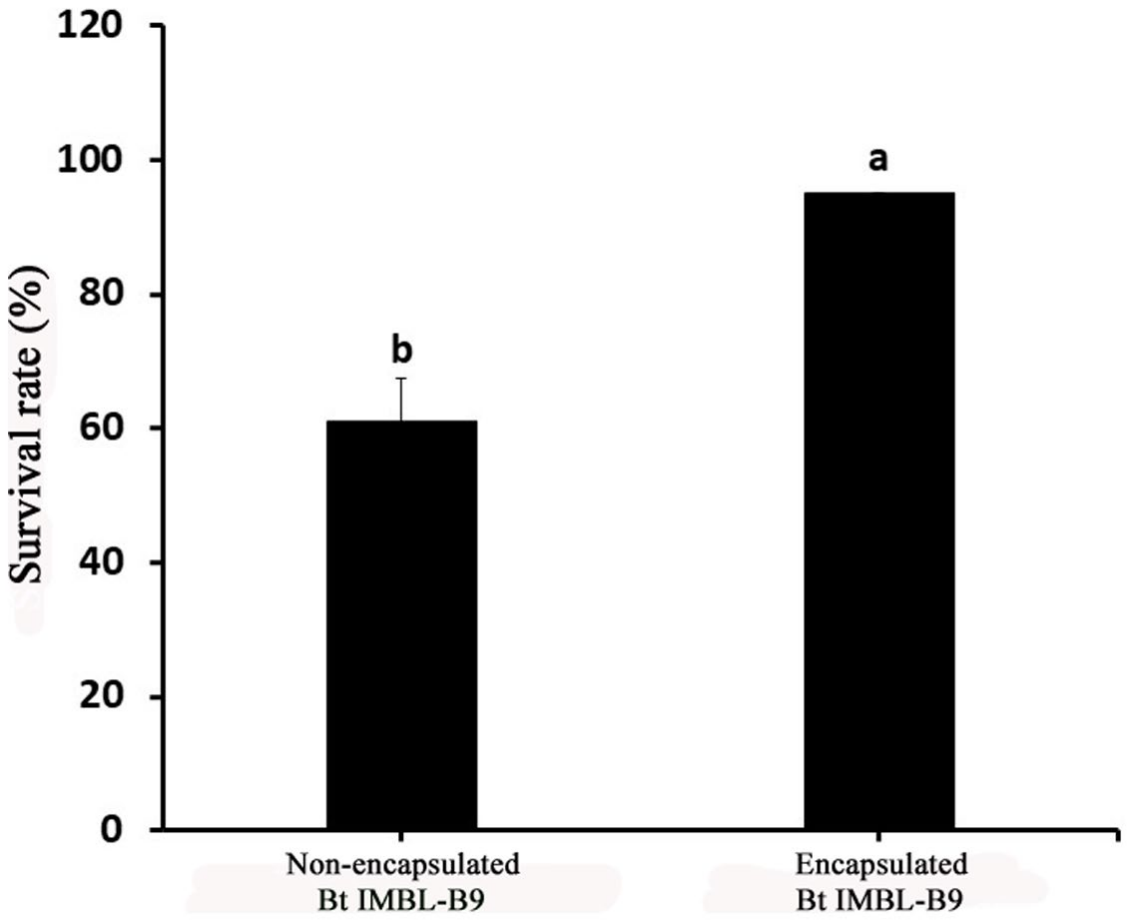
Survival rate of Bt-IMBL-B9 spores after microencapsulation via spray drying.

The number of Btk IMBL-B9 CFUs encapsulated in the mixture of gum arabic, maltodextrin, and corn starch was 2.02 × 10^10^ CFU g^−1^, subsequently presenting a 95% spore survival rate after spray drying. In comparison, the number of non-encapsulated CFUs following spray drying was 2.23 × 10^10^ CFU g^−1^, and showed a 61% spore survival rate (Figure 2). These results indicate that the mixture of gum arabic, maltodextrin, and corn starch do provide protection for endospores of Btk IMBL-B9 during spray drying.

**Figure 3:**
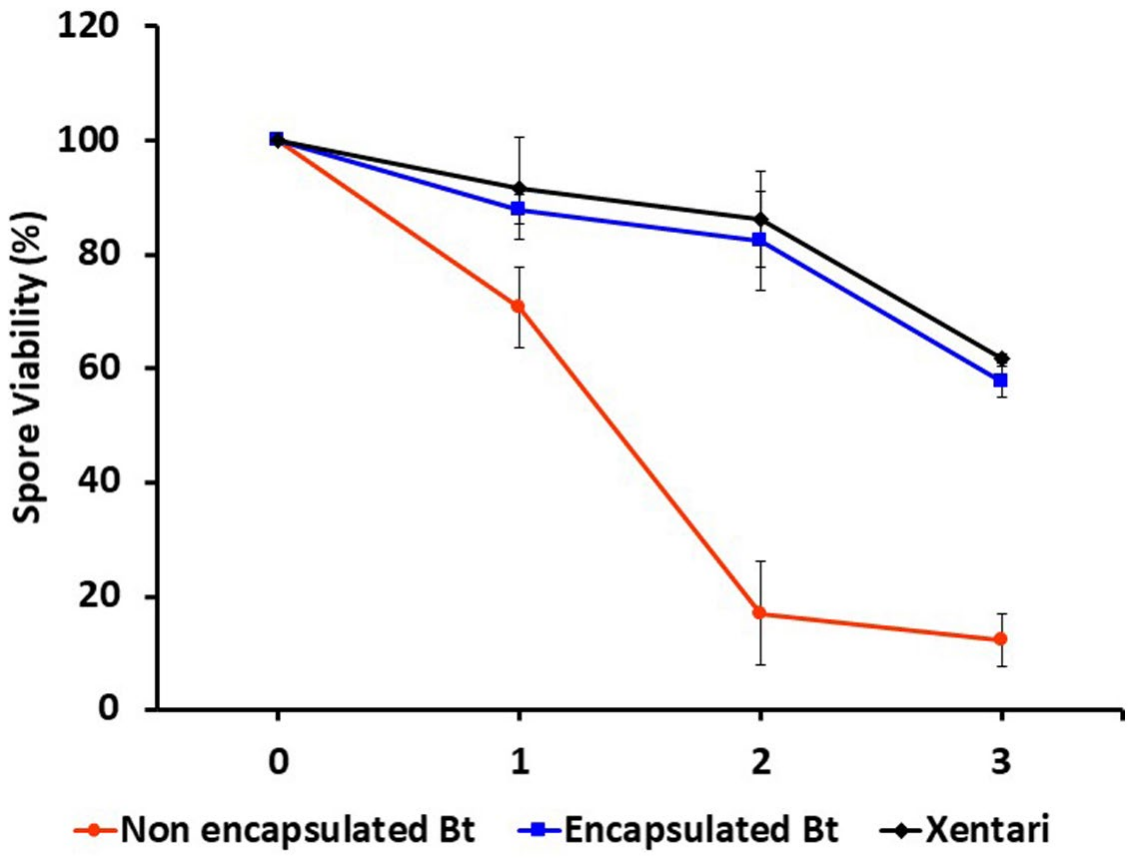
Spore viability curves by microencapsulation of Bt-IMBL-B9. Data are shown as the mean ± SE, derived from three replications.

In the interest of evaluating the impact of microencapsulation on the storage stability of Btk IMBL-B9, its viability was examined over a period of 6 weeks at 54 ± 2°C. At the end of this storage period, it was found that the spore viability of encapsulated Btk IMBL-B9 following spray drying was 57.6%, which is comparable with that of the commercial bioinsecticide XenTari^®^ (61.7% viability). In contrast, the non-encapsulated formulation of Btk IMBL-B9 showed only a 12.2% spore viability after 6 weeks in storage (Table 1; Figure 3).

**Table 1:** Stability of microencapsulation-based spray drying formulation of *Bacillus thurigiensis* subsp. *kurstaki* IMBL B9 formulations at different storage period at 54 ± 2°C.

Storage period (weeks at 54 ± 2°C)	Colony forming units (CFU g^−1^)		
	Non-encapsulated Btk IMBL-B9	Encapsulated Btk IMBL-B9	XenTari^®^
0	2.23 × 10^10c^	2.02 × 10^10c^	6.90 × 10^10a^
2	1.58 × 10^10b^	1.78 × 10^10bc^	6.31 × 10^10a^
4	3.78 × 10^9a^	1.67 × 10^10b^	5.95 × 10^10a^
6	2.72 × 10^9a^	1.16 × 10^10a^	4.26 × 10^10a^

Means in each column with the different lowercase superscript letters are significantly different (*p* < 0.05).

**Figure 4:**
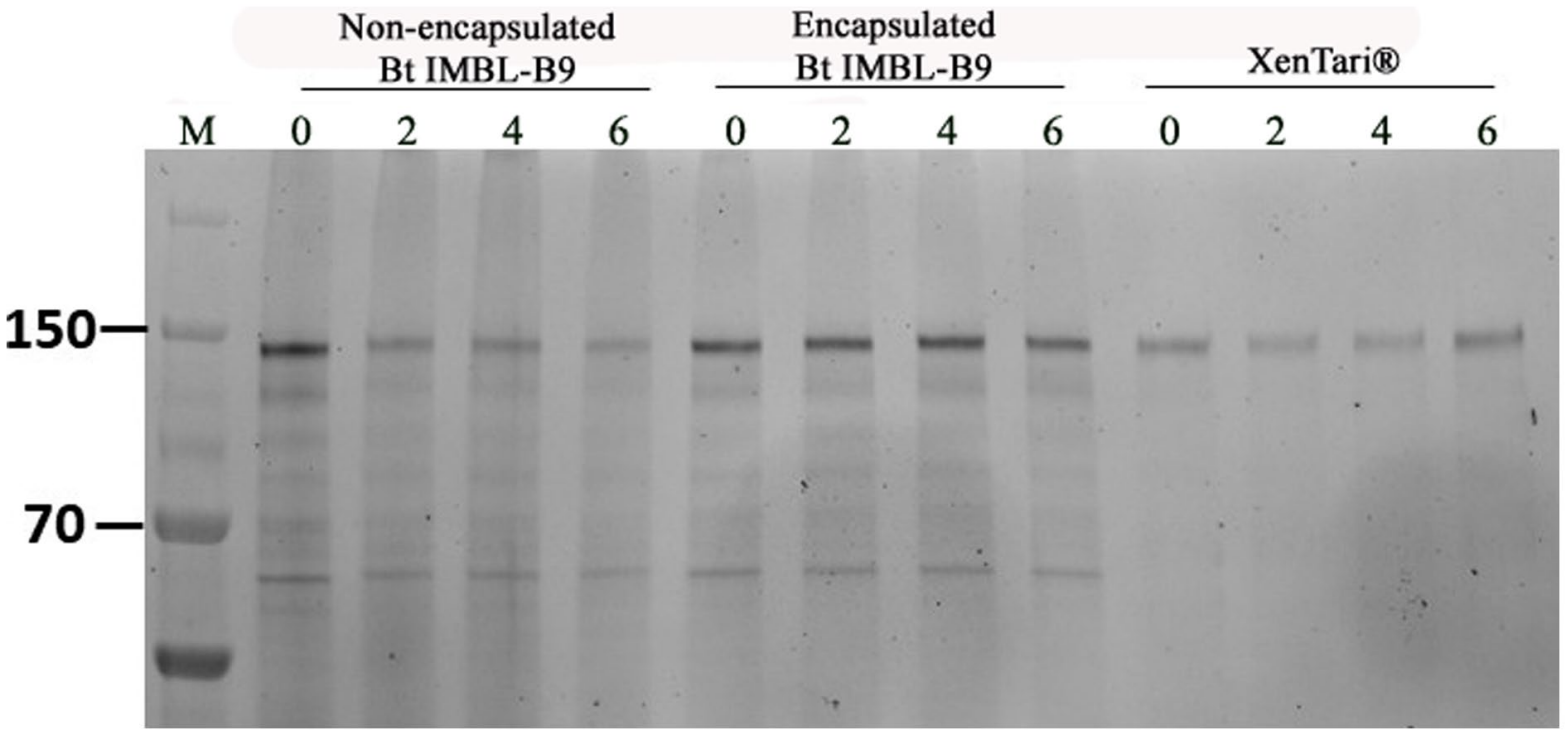
Cry protein SDS-PAGE of 1 mg mL^−1^ Bt formulations. Lane 0 ~ 6 means storage weeks at 54 ± 2°C.

The crystal protein content of encapsulated and non-encapsulated Btk IMBL-B9 samples and XenTari^®^ was analysed during a period of 6 weeks at 54 ± 2°C. In reference to results obtained through SDS-PAGE, it was found that the encapsulated formulation of Btk IMBL-B9 showed high-intensity bands, equating to molecular weights of 130 and 65 kDa. However, the intensity of the two bands from the non-encapsulated Btk IMBL-B9 sample was gradually weakened over the course of the 6 weeks. In comparison, that from XenTari^®^ was well maintained (Figure 4).

### Bioassay of encapsulated formulation of Btk IMBL-B9

**Figure 5:**
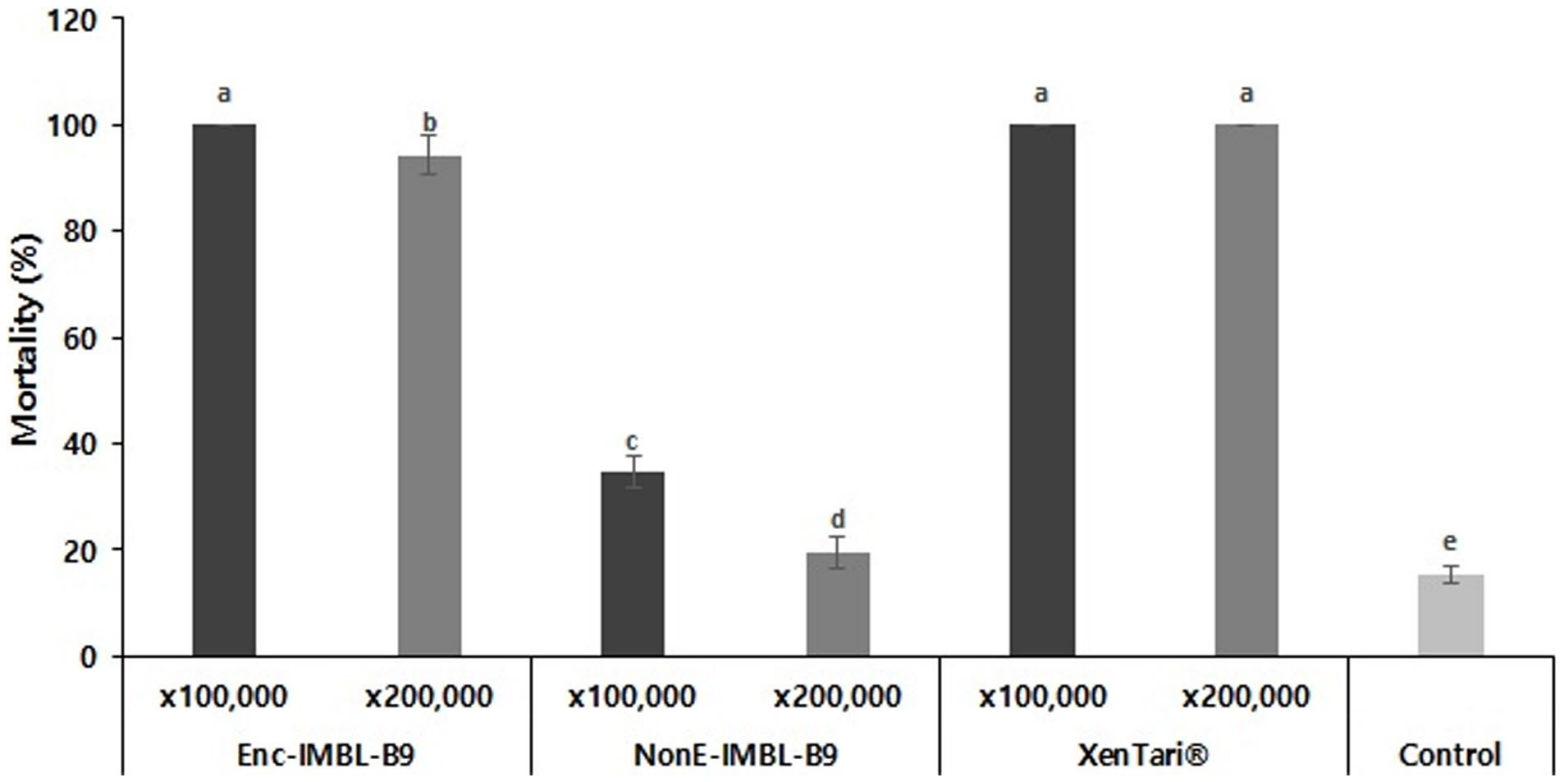
Insecticidal activity of encapsulated and non-encapsulated spray-dried formulations of IMBL-B9 stored at 54°C for 6 weeks against *P. xylostella* at 100,000- and 200,000-fold dilutions. XenTari^®^ stored at 54°C for 6 weeks was used as positive controls for bioassay. All assays were performed in triplicate and different letters (lower case) above the error bars indicate a significant difference by *post-hoc* test (*p* < 0.005).

After 48 h of treatment, the insecticidal activity of the encapsulated formulation of Btk IMBL-B9 stored at 54°C for 6 weeks against third-instar larvae of *P. xylostella* showed 100 and 94.3% mortality at 100,000- and 200,000-fold dilutions, respectively. The commercial bioinsecticide XenTari^®^ stored at 54°C for 6 weeks also showed strong insecticidal activity after 48 h of treatment, with 100 and 100% mortality at 100,000- and 200,000-fold dilutions, respectively (Figure 5). However, non-encapsulated IMBL-B9 sample which was stored at 54°C for 6 weeks showed very weak insecticidal activity against *P. xylostella*.

### Protection of intact plants treated with microencapsulation-based spray drying formulation of IMBL-B9 in the greenhouse

**Figure 6:**
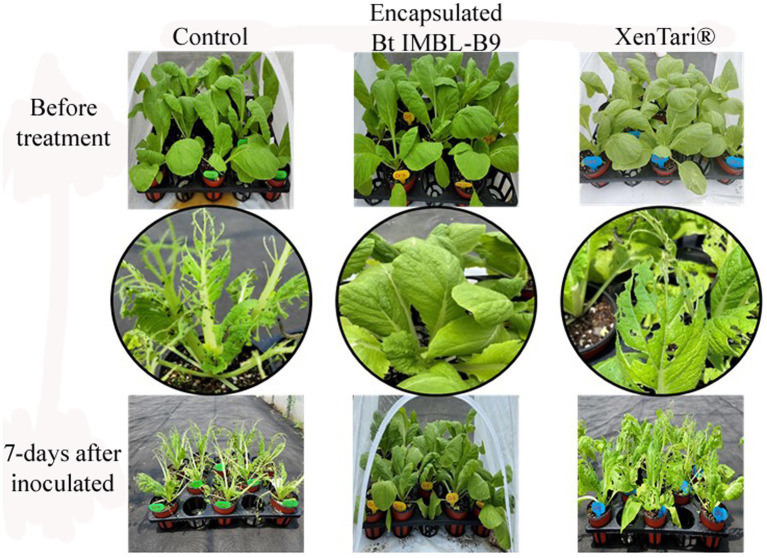
Chinese cabbage plants infested with third instar larvae of *P. xylostella* and *S. frugiperda* at 3 days after spraying with microencapsulation-based spray-dried formulation of Btk IMBL-B9. Distilled water was used as a negative control and XenTari^®^ as a positive control.

**Figure 7:**
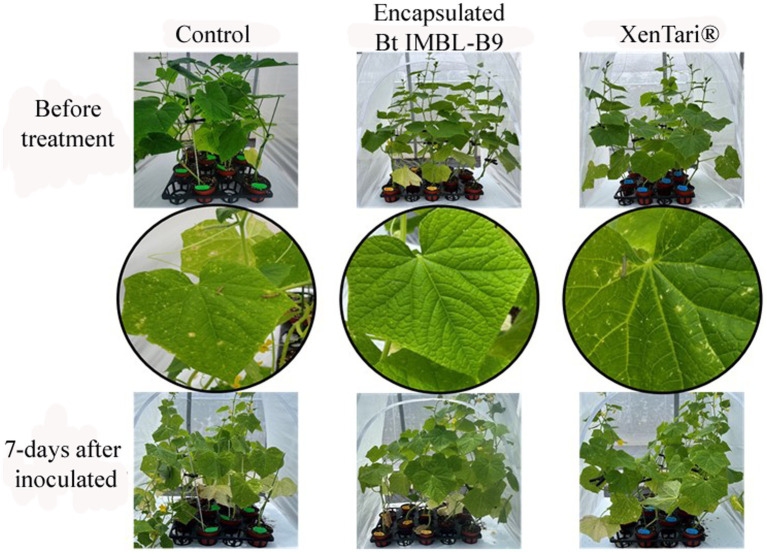
Cucumber plants infested with third instar larvae of *S. frugiperda* at 3 days after spraying with the microencapsulation-based spray-dried formulation of Btk IMBL-B9. Distilled water was used as a negative control and XenTari^®^ as a positive control.

To assess the insecticidal activity of the encapsulated formulation of Btk IMBL-B9 in a practical setting, greenhouse experiments were conducted against polyphagous pests, specifically *P. xylostella* and *S. frugiperda*. In the greenhouse study conducted on Chinese cabbage plants, it was evident that the insecticidal activity of the Btk IMBL-B9 formulation surpassed that of XenTari^®^, demonstrating its efficacy against both *P. xylostella* and *S. frugiperda* (Figure 6). In addition, *S. frugiperda* continued to attack the leaves of cucumber plants treated with XenTari^®^, resulting in scarring. However, the Btk IMBL-B9 formulation showed no signs of leaf damage, confirming its efficacy against pests on cucumber plants (Figure 7).

## Discussion

In a previous study, the Btk IMBL-B9 strain showed a high level of insecticidal activity against *P. xylostella, S. frugiperda,* and *S. exigua* (Park et al., 2022). However, an efficient formulation encompassing Btk IMBL-B9 is crucial for its successful use as an effective microbial insecticide. Bt can be formulated in several ways, including both liquid and dry preparations. Among the latter, spray drying is a versatile and efficient method for producing Bt formulations (Rosas-García, 2006; Yánez-Mendizábal et al., 2012; De Oliveira et al., 2021), as it is a relatively simple and cost-effective method for producing a large amount of Bt powder formulation (Santivarangkna et al., 2007).

The rough appearance commonly observed in spray-dried powders is due to rapid dehydration during the spray drying process (Nunes et al., 2018; Tao et al., 2019; Li et al., 2023). The morphology of spray-dried particles depends on the spray drying parameters: temperature, feed flow rate, and feed composition. When the drying process is conducted at low temperatures, slow water diffusion leads to the deformation and shrinkage of the material. Conversely, particles tend to inflate at high temperatures, forming a crust with occasional breakage due to rapid water evaporation and high internal pressure (Gutiérrez-López et al., 2015). Coating materials are considered protective agents which aid in the prevention of damage to the outer membranes of bacterial spores, which is caused by high temperatures during the spray drying process (Barajas-Álvarez et al., 2022).

No cracks were observed in microencapsulated spray-dried formulation of Btk IMBL-B9 particles, which denotes a high encapsulation efficiency and a 30% increase in spore viability following spray drying compared to non-encapsulated BTK IMBL-B9. Similar findings were presented for spray drying using gum arabic with maltodextrin (Arepally and Goswami, 2019; Barajas-Álvarez et al., 2022). In a previous study, the mixture of gum arabic and maltodextrin showed an 89% survival rate for the probiotic *Lactobacillus helveticus* after spray drying (Barro et al., 2021). This mixture also showed a high encapsulation efficiency for *L. rhamnosus* and *L. acidophilus* after spray drying (Arepally and Goswami, 2019; Barajas-Álvarez et al., 2022). In this study, we developed a new encapsulated formulation of Btk IMBL-B9 with a high spore survival rate using a mixture of gum arabic, maltodextrin, and corn starch. To the best of our knowledge, this is the first paper reporting a high encapsulation efficiency for Bt using this mixture.

Furthermore, the development of a stable formulation is crucial to extend the shelf life of Bt biopesticides effectively. This stability is vital for maintaining the viability of Bt over an extended period, ultimately enhancing its efficacy and practical applicability in pest management (Lacey et al., 2015). The storage stability of the microencapsulation-based spray-dried formulation of IMBL-B9 was similar to that of the commercial bioinsecticide XenTari^®^, but significantly more effective compared to that of the non-encapsulated Btk IMBL-B9 sample. These results indicate that the encapsulation of Btk IMBL-B9 with gum arabic, maltodextrin, and corn starch, significantly increases its protection and stability, thereby maximizing its effectiveness as a form of pest control.

Among various Bt crystal protein genes, it has been reported that *cry1Aa*, *cry1Ab*, *cry1Ac*, and *cry1B* are specifically active against *P. xylostella* (Entwistle et al., 1993). Conversely, *cry1C*, *cry1D*, *cry1E*, and *cry1F* have been reported to show an effectiveness against *Spodoptera* spp. (Chambers et al., 1991; Kalman et al., 1995; Valicente et al., 2010). Because the Bt subsp. *kustaki* HD-1 harbours *cry1Aa*, *cry1Ab*, and *cry1Ac* genes, this strain shows insecticidal activity against *P. xylostella,* but not against *Spodoptera* spp. (Kalman et al., 1995; Moar et al., 1995). Bt subsp. *aizawai,* which harbours *cry1Aa*, *cry1Ab*, *cry1C,* and *cry1D,* may be more effective against *S. frugiperda* than Bt subsp. *kurstaki* (Jepson et al., 2020; Bateman et al., 2021). In contrast to these findings, the Bt IMBL-B9 strain proved to be a broad-spectrum insecticide with high insecticidal activity due to its *cry* gene profile, which includes *cry1Aa*, *cry1Ac*, *cry1Be*, *cry1Ea*, *cry1Ha*, *cry2Aa*, *cry2Ab*, *cry2Ah*, and *vip3Aa* (Park et al., 2022).

In this study, the *in vitro* insecticidal activity of encapsulated formulation of Btk IMBL-B9 was significantly similar to that of XenTari^®^ against third-instar larvae of *P. xylostella.* However, it showed a much greater pest control efficacy than XenTari^®^ against *P. xylostella* and *S. frugiperda* on Chinese cabbage plants, as well as *S. frugiperda* on cucumber plants in greenhouse experiments. These results indicate that the encapsulated formulation of Btk IMBL-B9 can be widely utilized for the control of a broad spectrum of lepidopteran moths.

Collectively, the results of this study demonstrate that the microencapsulation process significantly improves the shelf life and stability of the parasporal crystals of Btk IMBL-B9. Furthermore, these findings suggest that the encapsulated formulation of Btk IMBL-B9 could be a promising environmentally-friendly and sustainable product for effective pest control. There is also immense potential for this novel Bt insecticide in large-scale production and efficient use in agriculture.

## Data availability statement

The original contributions presented in the study are included in the article/supplementary material, further inquiries can be directed to the corresponding author.

## Author contributions

KD: Conceptualization, Data curation, Methodology, Software, Writing – original draft. NY: Conceptualization, Writing – original draft, Funding acquisition. SK: Writing – original draft, Methodology, Investigation. JB: Methodology, Investigation, Writing – original draft. JS: Methodology, Investigation, Writing – original draft. EC: Funding acquisition, Writing – original draft. MP: Methodology, Investigation, Writing – original draft. JK: Methodology, Investigation, Writing – original draft. JC: Methodology, Investigation, Writing – review & editing. MS: Methodology, Investigation, Writing – original draft. YJ: Writing – review & editing. J-CK: Conceptualization, Writing - review & editing.
